# Treatment of Girolando cattle dermatophilosis using a combination of different plant extracts in the municipality of Abomey-Calavi, Republic of Benin

**DOI:** 10.14202/vetworld.2021.2750-2756

**Published:** 2021-10-25

**Authors:** Camus Adoligbe, Ricoland Gangbe, Justin Adinci, Samuel Mantip, Souaïbou Farougou

**Affiliations:** 1 Research Unit on Communicable Diseases, Polytechnic School of Abomey-Calavi, University of Abomey-Calavi, Cotonou, 01BP 2009, Benin; 2 Virology Division, National Veterinary Research Institute. PMB 0001, Vom, Nigeria.

**Keywords:** farmer resilience, indigenous plants, plant extract, sustainable cattle breeding

## Abstract

**Background and Aim:**

Dermatophilosis is a bacterial infection of the skin of animals. It is prevalent worldwide and is caused by *Dermatophilus congolensis*. The study aimed to assess the therapeutic efficacy of different mixtures prepared with indigenous phytogenetic extracts from Benin in the management and treatment of Girolando cattle that showed high sensitivity to the disease compared to any other known cattle breed in Benin.

**Materials and Methods:**

Consequently, two types of extact mixtures (extract mixture 1=*Elaeis guineensis* kernel oil + essential oil of *Ocimum gratissimum* + sap of *Jatropha multifidi;* extract mixture 2*= O. gratissimum* + sap extract of *J. multifida*) of 20% concentration were prepared and tested on eight Girolando cattle. Two different parts of the affected skin with acute lesions of dermatophilosis were debrided, and 0.5 mL of each of the extract mixture was applied per square centimeter of a single zone of the affected skin.

**Results:**

Both extracts mixtures were found to possess significant wound healing properties compared to the control (procaine G penicillin). However, the extract mixture 1, which was made up of *Elaeis guineensis* kernel oil, essential oil of *O. gratissimum* and sap of *J. multifida* (Linn), showed a better result. This was evident by increase in the rate of wound contraction and healing without recurrence 2 weeks after the end of the experiment and the subsequent immediate manifestation of hair or hair growth at the affected area.

**Conclusion:**

The preliminary findings of this study are very promising. Extract mixture 1 could serve as an alternative in the treatment or management of bovine dermatophilosis in Benin and other dermatophilosis endemic areas of the world. However, *in vitro* testing and sensitivity against isolated *D. congolensis* organism using extract mixture 1 as well as cost implications should be studied.

## Introduction

The agricultural sector is one of the major economic drivers in Benin, contributing 22.6% to the country’s gross domestic product (GDP) [1]. It creates employment and food security for the teaming population; it also contributes to industrial and rural development. The production of livestock (cattle) is important in the agricultural sub-sector, and it contributes about 17.2% to the agricultural GDP in Benin [2]. Cattle are the major producers of animal protein (58%) in the livestock sub-sector, followed by poultry (21%), small ruminants (13%), and pigs (7%) [3,4].

As part of the revitalization plan of the agricultural sector in Benin by the government, efforts have been put on-ground to boost the production of indigenous breeds through crossbreeding with high producing improved breeds of cattle. Therefore, Girolando dairy cattle known to be a crossbred product of Holstein and Gir, respectively, were imported from Brazil into the country to improve the milk production performance of local breeds of cattle in the country [5].

However, imported animals and their crossbred products from the temperate regions of the world, mostly find it difficult to favorably thrive in the African tropical region due to the hot and harsh environmental climate. Other challenges that militate against their production include attack by endemic infectious and contagious diseases, which include dermatophilosis among other devastating diseases.

The distribution of Bovine dermatophilosis is worldwide, but it is mostly recorded in tropical African countries, including Benin [6,7]. It is a tick-borne disease caused by an actinomycetes bacterium, *Dermatophilus congolensis*, characterized by an exudative acute or chronic dermatitis that could be localized or generalized [8,9]. The lesions vary in size and severity, from small lesions (small paintbrush-like) and clear circumscribed scabs to more confluent progressive lesions. The disease results in gradual loss of body condition, decrease in milk and meat production, reduced working ability in draft animals, poor reproductive performance, decreased economic values of hide and skin, and losses due to mortality in weak animals [10]. In tropical areas, animals that recover from the wound caused by the disease were reported to be re-infected during the successive wet season, indicating the endemicity of the disease in the country [11]. It has been recorded that a wide variety of drugs are being administered with little or poor efficacy to the disease [12]. In addition, it has also been reported that the treatment and management of the disease using chemo-therapy or pharmaceutical intervention have not been productive in suppressing the severity of the lesions over the years [13], and it has been reported that disseminated lesions are generally refractory to therapy. In Benin, Ali-Emmanuel [14] showed that ointments made with the ethanolic extracts of leaves of *Senna alata*, *Lantana camara*, or *Mitracarpus scaber*, when applied once a day for 8-15 days, the effect of the mixture which stimulates the falling off of the crusts could start manifesting as early as by 3-4 days of application and the total healing of the disease could subsequently occur by 3-4 weeks after the end of the treatment. The selected ethnoveterinary phytogenetic extracts, which include *Ocimum gratissimum*, *Jatropha multifida* (Lin), and *Elaeis guineensis* (kernel oil), have been proven over the years to have antibacterial/anti-inflammatory properties [15-17]*. O. gratissimum* leaves have been used in traditional medicine as a general tonic and in the treatment of diarrhea and conjunctivitis in humans [18]. The oil extracted from the leaf of *O. gratissimum* mixed with alcohol has been applied as a lotion against skin infections and has also been taken orally to treat bronchitis. The extract from the dry leaves, when boiled and administered orally, has been used to relieve headache and fever among other ethnomedicinal uses in humans [19]. The essential oil of this plant extract has a remarkable antibacterial effect, superior to those of commercial anti-septic pharmaceutical products when applied topically on the skin by humans [20]. It also regulates the nervous system, stimulates digestion and relieves osteoarthritis in humans. *J. multifida* (Linn) is locally called “five fingers plant” in Benin [21]. It is a plant of the Euphorbiaceae family with a widely recognized medicinal property. It was found to be helpful in the management and treatment of various diseases by nearly 80% of the human population in Africa, Asia, and Latin America [22]. *J. multifida* (Linn) is known over the years as one of the medicinal plants whose sap extract has been widely used for wound healing in Benin. Agban *et al*. [23] and Aiyelaagbe *et al*. [24], have previously reported the antibacterial effect of the leaves, stems, and roots extract of *Jatropha multifida* Linn. Indeed, *E. guineensis* (kernel oil) extract has been used over the years in Nigeria in the treatment and management of various skin diseases and infections in both humans and animals [25].

The study aimed to assess the therapeutic efficacy of different mixtures prepared with indigenous phytogenetic extracts from Benin in the management and treatment of Girolando cattle that showed high sensitivity to the disease compared to any other known cattle breed in Benin.

## Materials and Methods

### Ethical approval

The plant products used in this study were common traditional medicinal plants that are widely used in the management and treatment of some common human ailments in Benin without any formal government regulation. For this reason, this study was conducted in complete agreement with the Benin national veterinary and pharmaceutical regulations. Therefore, no ethical approval was necessary.

### Study period and location

The study was conducted during September and October 2020. The experimental study was carried out in a semi-intensive ruminant farm located in the municipal area of Abomey-Calavi, Department of Atlantic, Benin. Abomey-Calavi area is characterized by a sub-equatorial climate of four seasons that alternates two rainy seasons and two dry seasons. The two rainy seasons run from March to July and from September to November, respectively, while the dry seasons extend from July to September and from December to March, respectively. The average temperature in Benin is between 28 and 29°C, with rainfall ranging from 1100 to 1300 mm. The geographical aerial view shows Plateaus dominating the entire region with a maximum altitude of 31 m above sea level. Agriculturally, the vegetation is heterogeneous in nature, characterized by savannah shrubs, tropical short trees, and lush grass cover [26].

### Animals

**Figure-1:**
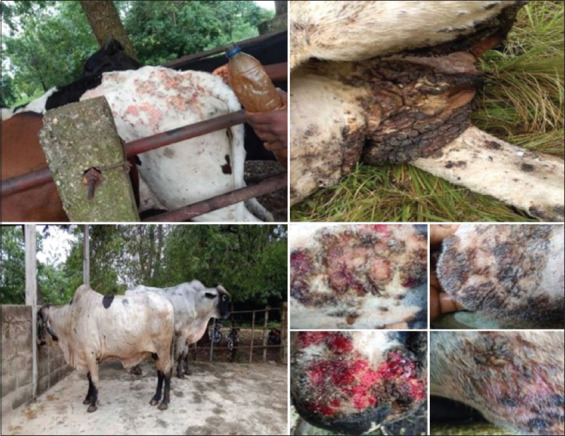
A live Girolando cattle and dermathophilosis lesions.

Nine Girolando cattle presenting acute lesions of dermatophilosis as described previously [12,14] were used in the experiment (Figure-1). To circumvent the influence of external factors, the animals were not allowed to pasture outside the paddock during the trial. They were fed in the cowshed and routine farm prophylaxis was strictly followed.

### Essential oil extract of *O. gratissimum*

The essential oil extract of *O. gratissimum* was obtained by hydro-distillation [27] from the leaves of *O. gratissimu*m harvested during the month of September near Abomey-Calavi. The plant was identified and authenticated by Dr. Yedomonhan H. from the National Herbarium of the University of Abomey-Calavi. Voucher specimens were deposited in the National Herbarium of the University of Abomey-Calavi in the Republic of Benin under the number (YH640/HNB). The harvested leaves were dried for 3 days under a shade area and the oil was extracted at the Applied Chemistry Research Laboratory of the University of Abomey-Calavi using a Clevenger extraction apparatus (MH1201, Maihun, Jiangsu, China). The leaves were put in a flask containing boiled (100°C) water. The vapor rises in the assembly of the flask to a condensed level, and then the condensate in liquid form was allowed to freely drip into a small beaker. The oil extract eventually floats on the liquid and was gradually returned to the heated flask through the diagonal conduit. After 2 h of extraction, the volume of the oil extract collected in the burette was directly measured and then stored in the refrigerator at 4°C during the experimentation time.

### Sap extract of *J. multifida* (Linn)

The sap extract of *J. multifida* (Linn) was collected from a fresh Linn stem during the month of September 2019 in the municipal areas of Abomey-Calavi and Allada. The plant was identified and authenticated by Dr. Yedomonhan H. from the National Herbarium of the University of Abomey Calavi. Voucher specimens were deposited in the National Herbarium of the University of Abomey-Calavi in the Republic of Benin under the number (YH639/HNB). The extract was carefully collected directly into the Eppendorf tubes after cutting the stem while taking care to avoid external contamination [17]. The harvested samples were kept at 4°C in the refrigerator during the experimentation time.

### Oil extracts of *E. guineensis* (kernel oil)

Raw black *E. guineensis* (kernel oil) extract was purchased from the market and filtered to remove palm nut residue.

### Determination of the optimal concentration of the mixture component

To determine the optimal concentration of the products to be mixed, eight zones of 5 cm² of infected areas were each debrided on the infected skin of the sick animals and different concentrations of the products were tested. 0.5 mL of each of the extracted products was applied independently per square centimeter of the infected areas. The application of the different extracts was carried out in every 2 days’ interval for 10 consecutive days. At the end of the pre-experimental phase, 20% concentration of each of the extracts turned out to be the most efficacious for each of the solutions and was thereafter retained for application for the rest of the experiment (Table-1).

**Table-1:** Optimal concentration determination for each product.

Zone	Phytogenetic resources/extracts	Concentration (%)	Quantity (mL)	Application area (cm²)
1	Acetone (additive acting as an emulsifier)		2.5	5
2	PKO		2.5	5
3	EOOG	5	2.5	5
4	EOOG	10	2.5	5
5	EOOG	20	2.5	5
6	SJML	5	2.5	5
7	SJML	10	2.5	5
8	SJML	20	2.5	5
9	Negative control			5

EOOG=Essential oil extract of *Ocimum gratissimum*, SJML=Sap extract of *Jatropha multifida* Linn, PKO=*Elaeis guineensis* kernel extract.

### Mixture preparation

During the combination procedure, two extracts mixtures were prepared based on the selected phytogenetic extracts and were tested on the lesions of the dermatophilosis infected animals (Table-2). These include the mixture of equal volume (33 mL) of essential oil extract of *O. gratissimum*, sap extract of *J. multifida* (Linn) and raw black *E. guineensis* (kernel oil) extract (Extract mixture 1) and mixture of half volume (50 mL) of essential oil extract of *O. gratissimum* and sap extract of *J. multifida* (Linn) (Extract mixture 2).

**Table-2:** Mixture preparation of phytogenetic extracts for the treatment of dermatophilosis infected animals.

Products	EOOG (20%)	SJM (20%)	PKO
Mixtures			
Extract mixture 1	33.33%	33.33%	33.33%
Extract mixture 2	50%	50%	0%

EOOG=Essential oil extract of *Ocimum gratissimum*, SJML=Sap extract of *Jatropha multifida* Linn, PKO=*Elaeis guineensis* kernel extract.

### Mixture application

The efficacy of the mix or combined extracts was tested on 8 out of 9 selected animals; two affected zones on the skin presenting crusty lesions were debrided. Each of the extract mixtures (2.5 mL) was applied to a specific infected zone (5 cm^2^) on each of the animals every 48 h for 19 consecutive days. The hard crusts were removed from all the infected zones before the application of the extracts mix. The ninth animal was used as a control and was receiving the medication used on the farm previously (Intramuscular injection of penicillin G procaine every 2 days at a dosage of 1 mL/20 kg body weight). Four infected zones on the skin presenting crusty lesions of dermatophilosis were debrided on the control animal to assess the wound healing progress.

### Wound healing assessment

The progress of the wound healing over the period of the experiment was used as an indicator to assess the therapeutic efficacy of each of the mixtures compared to the control in the treatment of dermatophilosis on the cattle used for the experiment. The infected wound areas on the skin were measured by tracing the wound margin using a tape measured on day 1 of the experiment and subsequently on every 2 days until day 19. The healed areas were calculated by subtracting the real-time wound area from the original wound area. The percentages of wound contraction were calculated using the formula: Percentage of wound contraction=(Healed area/Total wound area) [28]. The average percentage of wound contraction induced by each of the mixtures was obtained by dividing 8 by the sum of the percentage of wound contraction obtained from each of the respective extract mixture application zones. The average percentage of wound contraction induced by penicillin G procaine was obtained by dividing the sum of the percentage of wound contraction obtained from each of the four targeted zones.

### Statistical analysis

Data are expressed as mean±standard deviation. Results were analyzed by the Generalized Linear Models (Proc GLM) procedure followed by Student’s t-test using statistical analysis system software R version 3.5.1 (R Foundation for Statistical Computing, Vienna, Austria) [29].

## Results

### Wound contraction progress

**Figure-2:**
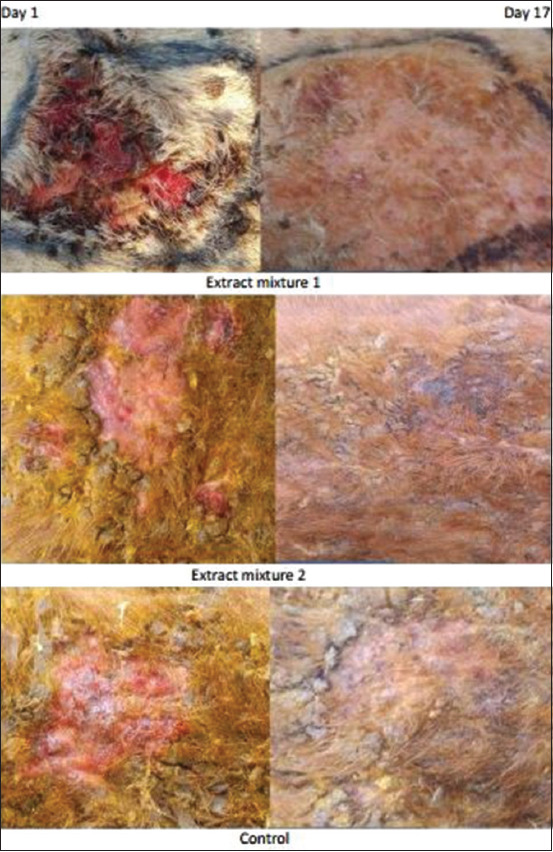
Wound healing progress of dermathophilosis over experimental time with the application of different mixtures.

The progress of wound contraction in each group over the experimental time is shown in Figure-2 and Table-3. Overall, we observed a progressive positive trend of average wound contraction during all the days of the experiment. However, statistically significant average wound contraction was exhibited among groups from day 9 to day 19 (Table-3). Average wound contraction was significantly better in Group 1 than the control group from day 7 to day 13 (p<0.05), and very significantly better in Group 1 than the control group and Group 2 from day 15 to day 19 (p<0.01). As shown in Figure-2, hairs start growing on the treated areas, which eventually healed with few scarring on day 17 (Extract mixture 1).

**Table-3:** Average percentage of wound contraction at various experimental days.

Time (Day)	Experimental Groups			p-value

	Control	Group 1	Group 2	
3	18.06±6.49a	31.59±3.31a	31.47±6.51a	0.1068
5	16.79±5.40a	24.84±15.28a	31.47±6.51a	0.09251
7	22.74±3.94a	34.84±12.62a	39.86±15.30a	0.09213
9	29.86±4.26b	57.51±5.85a	50.85±4.91a	0.01089
11	30.67±4.24b	64.82±5.69a	54.24±5.38a	0.002191
13	34.38±2.37b	68.40±7.82a	54.32±5.42a	0.003773
15	41.55±1.29c	87.71±1.81a	59.74±9.96ab	0.0001875
17	47.51±2.46c	100±0.18a	65.61±3.36b	0.0003338
19	53.07±15.38c	100±018a	72.75±3.13b	0.0006247

Group 1: Zones treated with extract mixture 1 (EOOG+SJML+PKO), Group 2: Zone treated with extract mixture 2 (EOOG+SJML); Mean+standard deviation within rows with different letters are significantly (p<0.05, p<0.01or p<0.001) different according to Student’s t-test

## Discussion

Dermatophilosis in cattle is characterized by exudative dermatitis with scab formation. The previous scholars have shown that ethnoveterinary approaches are preferred in wound healing or cure of the disease since they are devoid of side effects and are more effective [30,31]. The present study aims at mixing or diluting different phytogenetic extracts with proven records of antibacterial/anti-inflammatory activity in both humans and animals. These phytogenetic extracts include; essential oil extract of *O. gratissimum*, sap extract of *J. multifida* (Linn), and raw black Palm kernel extract for topical treatment of bovine dermatophilosis lesions in Benin. The outcome of our findings indicated that each of the tested extract mixtures has a wound-healing capability, but at a different rate with extract mixture 1, which contains (3 phytogenetic extracts) that are considered the most efficacious substance against bovine dermatophilosis. This variation in wound healing effect could probably be due to the different chemical components of each of these phytogenetic extracts.

It has been previously stipulated that oxidative stress has been associated with most acute and chronic inflammatory conditions such as wound healing, but specifically, flavonoid component of the plant extracts was also known to be responsible for free radical scavenging, which is important in wound healing [28]. The previous study that was conducted in southern Benin on the chemical profile of the sap extract of *J. multifida* (Linn) revealed the presence of flavonoids, tannins, and terpenes groups [32]. Many scholars have previously proved the wound healing activity of these chemical compounds. For instance, Sheeba *et al.*, [33] and Mondal and Suresh [34] have previously reported that flavonoids and tannins were present in *Cleome rutidosperma*’ roots and *Cassia occidentalisa* leaves, and they observed healing of experimentally induced wounds in rats. Besides, the antioxidant and antimicrobial activities of flavonoids contained in the sap extract of *J. multifida* (Linn) have also been reported [23,35,36].

The previous analysis of essential oil extract produced from the two common types of *O. gratissimum* grown in Benin has shown the presence of three main components, which includes thymol, p-cymene, and γ-Terpinene, though; their concentration may vary under the influence of different factors [37]. The antimicrobial, anti-inflammatory, and rapid wound healing activities of the essential oil extract of *O. gratissimum* have been previously attributed to the presence of these components [15,38].

On the other hand, *E. guineensis* (kernel oil) extract is also commonly known to be used in South-Eastern Nigerian communities over the years to treat various skin diseases in humans and animals [25]. Mboui [16] reported that the mineral components of *E. guineensis* (kernel oil), particularly zinc, induced complete wound healing after 2 weeks of daily application.

We then strongly believe that based on the outcome of our findings, the following stated rationale was behind the assumptions that suggest plant products give better results than chemotherapeutic agents in wound healing [39]. In addition, Klotoé *et al*. [32] have also previously reported a complete wound healing activity of the sap extract of *J. multifida* (Linn) in rats and grass-cutter after 18 and 19 days of treatment, respectively. Ointments made with the ethanolic extracts of leaves of *S. alata*, *L. camara*, and *M. scaber* have also been used as topical treatments on chronic crusty or acute lesions of dermatophilosis with very good results [14]. Consequently, based on the outcome of their findings, the ointments used in their experiment practically stimulate the detachment and falling off of the crusts after 3-4 days of treatment when applied once a day for 8-15 days [14]. Hair production and growth then commence immediately on the treated side, which heals without scarring within 3-4 weeks after the end of the treatment. In this preliminary experimental trial, the plant extracts mixture application was performed every 2 days, and complete wound healing was attained on the 17^th^ day of treatment. The results obtained were almost conclusive and could even be much better if the extract mixture application was performed on a daily basis.

## Conclusion

As a preliminary study, the findings of this present study demonstrate that a mixture of essential oil extract of *O. gratissimum*, sap extract of *J. multifida* (Linn), and raw black *E. guineensis* (kernel oil) extract promotes skin wound healing activity in cattle and therefore constitute an alternative remedy for bovine dermatophilosis lesion treatment. We, therefore, recommend further study such as *in vitro* testing and sensitivity against isolated *D. congolensis* organism using extract mixture 1 be conducted to comprehend the complete wound healing mechanism of the extract mixture and that cost implications be assessed.

## Authors’ Contributions

CA: Designed the study, drafted, critically revised the manuscript, and collected samples. RG: Designed and managed the study, and wrote the manuscript. JA: Participated in the design of the study and interpretation of the data. SM: Revised and edited the manuscript. SF: Revised and approved the final manuscript. All authors read and approved the final manuscript.
